# Delayed Lower Extremity Monoplegia After Anterior Cervical Discectomy and Fusion: A Report of a Rare Case of Cervical Spinal Ischemic Reperfusion Injury

**DOI:** 10.7759/cureus.65071

**Published:** 2024-07-22

**Authors:** Rose V Zach, Mohamed Abdulhamid, Navid Valizadeh, Victor Zach

**Affiliations:** 1 Medicine, University of Arizona College of Medicine - Phoenix, Phoenix, USA; 2 Neurological Surgery and Spine Surgery, Royal Spine Surgery, Phoenix, USA; 3 Neurology, University of Texas at Austin Dell Medical School, Austin, USA; 4 Osteopathic Medicine, Midwestern University Arizona College of Osteopathic Medicine, Glendale, USA; 5 Osteopathic Medicine, A.T. Still College of Osteopathic Medicine, Mesa, USA

**Keywords:** neurosurgery, neurology, neurotrauma, anterior cervical discectomy fusion, white cord syndrome, ischemic-reperfusion, cervical spine

## Abstract

White cord syndrome is an extremely rare type of cervical spinal cord ischemia characterized by sudden neurological deterioration following surgical spinal decompression. The underlying cause is believed to be immediate relief from chronic compression on the spinal cord, triggering an inflammatory response known as ischemic reperfusion injury. A 49-year-old male presented in the office with neck pain and chronic symptoms of progressive cervical myelopathy: clumsiness, gait instability, and dropping things. An MRI of the cervical spine demonstrated severe central canal stenosis with spinal cord compression and myelomalacia at the C3-C4 level. The patient underwent a planned anterior cervical discectomy and fusion procedure from C3 to C5. Following the surgery, he developed monoplegia in his left lower extremity. His postoperative MRI revealed white cord syndrome, characterized by an increase in the signal change of the spinal cord. This finding was consistent with an ischemic reperfusion injury to the spinal cord post-decompression. White cord syndrome is thought to be caused by a reperfusion injury following surgical decompression of a previously compressed segment of the spinal cord, characterized by the rapid return of blood flow. This involves oxidative damage caused by free oxygen radicals and inflammatory molecules, such as reactive oxygen species, which lead to lipid peroxidation of neuronal membranes. Surgeons should be aware of this rare complication and warn patients preoperatively.

## Introduction

Anterior cervical discectomy and fusion (ACDF) is a commonly performed surgical procedure for cervical spine conditions and has generally shown positive clinical outcomes [1]. However, one of the most serious complications that can occur after cervical spine surgery is the development of neurologic deficits, such as paralysis or paraplegia, below the cervical level at which the procedure was performed. Neurological decline following cervical spine decompression surgery is typically due to iatrogenic injury, compressing hematoma, or hardware failure [2]. Nevertheless, there are instances where paraplegia occurs without a clear explanation, and in such cases, reperfusion injury, known as “white cord syndrome,” may be the underlying cause. Cervical spinal ischemic reperfusion injury (CSIRI) is an extremely rare complication that arises after decompression surgery, typically ACDF. White cord syndrome is an exceptionally rare condition, and limited information is available in the existing literature regarding this condition. The underlying cause is believed to be immediate relief from chronic compression on the spinal cord, triggering an inflammatory response known as ischemic reperfusion injury. The precise pathophysiology of this injury is not fully understood, but it is postulated to occur through various mechanisms, as follows [3,4]: First, the rapid return of blood flow to compressed areas of the spinal cord is believed to cause injury. Additionally, it is postulated that oxidative damage by free oxygen radicals may contribute to spinal cord injury once blood flow is reestablished. Lastly, inflammatory molecules such as reactive oxygen species lead to lipid peroxidation of neuronal membranes, contributing to damage. Postoperative T2-weighted MRI indicating white cord syndrome has shown increased signal intensity within the spinal cord, indicating cord ischemia and edema [4,5]. This study provides a review of the literature and presents an additional case of this complication following an ACDF procedure. This abstract was presented at the American Society of Neuroimaging Annual Meeting in Scottsdale, Arizona, United States, on August 12, 2023. This case was presented virtually at the 9th World Congress on Spine and Spinal Disorders in Dubai, UAE on February 1-2, 2024.

## Case presentation

A 49-year-old male with neck pain and dizziness was evaluated in the office. Five months prior to the visit, he underwent an industrial injury in which a 400-pound (approximately 181 kilograms) wooden beam fell roughly 25 feet (approximately 7.6 meters) onto the patient’s head. He complained of headaches, neck pain with stiffness, nausea, vomiting, memory problems, and “dizziness.” He was diagnosed with a mild traumatic brain injury without loss of consciousness.

The patient had a past medical history of hyperlipidemia, hypertension, and lipoma resection. His subjective symptoms included neck pain with a Visual Analogue Scale of 10/10 and a Neck Disability Index of 43/50, stiffness with severely limited range of motion in all directions, and heaviness (Appendices A, B). He experienced myelopathic symptoms, including gait instability, changes in dexterity, hand numbness, and dropping objects from his hands. He scored 13 (moderate severity) on the modified Japanese Orthopedic Association (mJOA) scale (Appendix C).

His objective signs included a BMI of 47.2, tenderness to palpation in the cervical region, limited range of motion of the cervical spine, non-dermatomal numbness of the left arm, and a positive Hoffman’s sign bilaterally. He was found to have cervicalgia with cervical spinal stenosis with spinal cord compression and myelomalacia at the C3-C4 and C4-C5 levels. Considering the specific characteristics of his cervical condition, which included severe stenosis with compression of the spinal cord and myelomalacia detected on the cervical spine MRI, along with his symptomatic presentation, the patient was recommended to undergo an ACDF procedure targeting the C3-C4 and C4-C5 levels.

The patient underwent the procedure without intraoperative complications or hypotensive episodes. Intraoperative neuromonitoring was performed and revealed improvements in relevant signals compared to baseline motor-evoked potentials of the patient’s extremities. The immediate PACU evaluation showed no focal neurological deficits.

**Figure 1:**
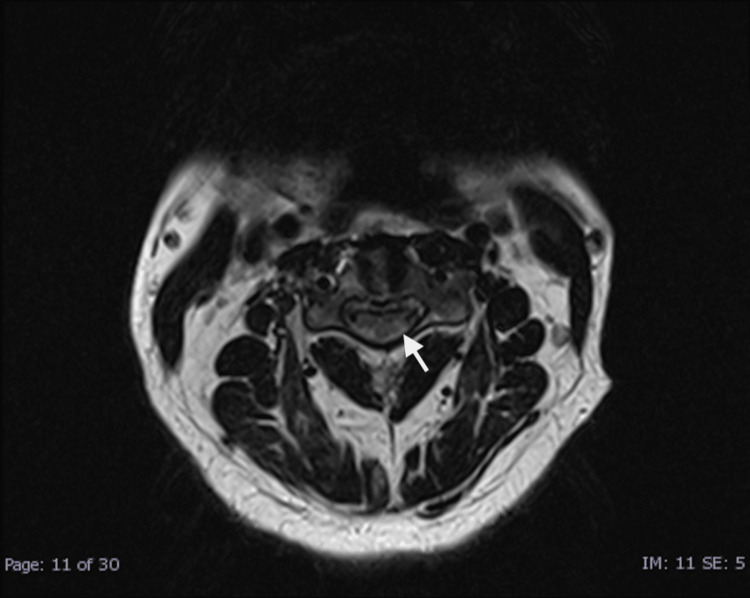
Axial T2-Weighted Image Axial T2-Weighted MR image of the spinal cord shows hyperintense signal change, indicative of ischemic spinal cord sprinkling and white cord syndrome.

**Figure 2:**
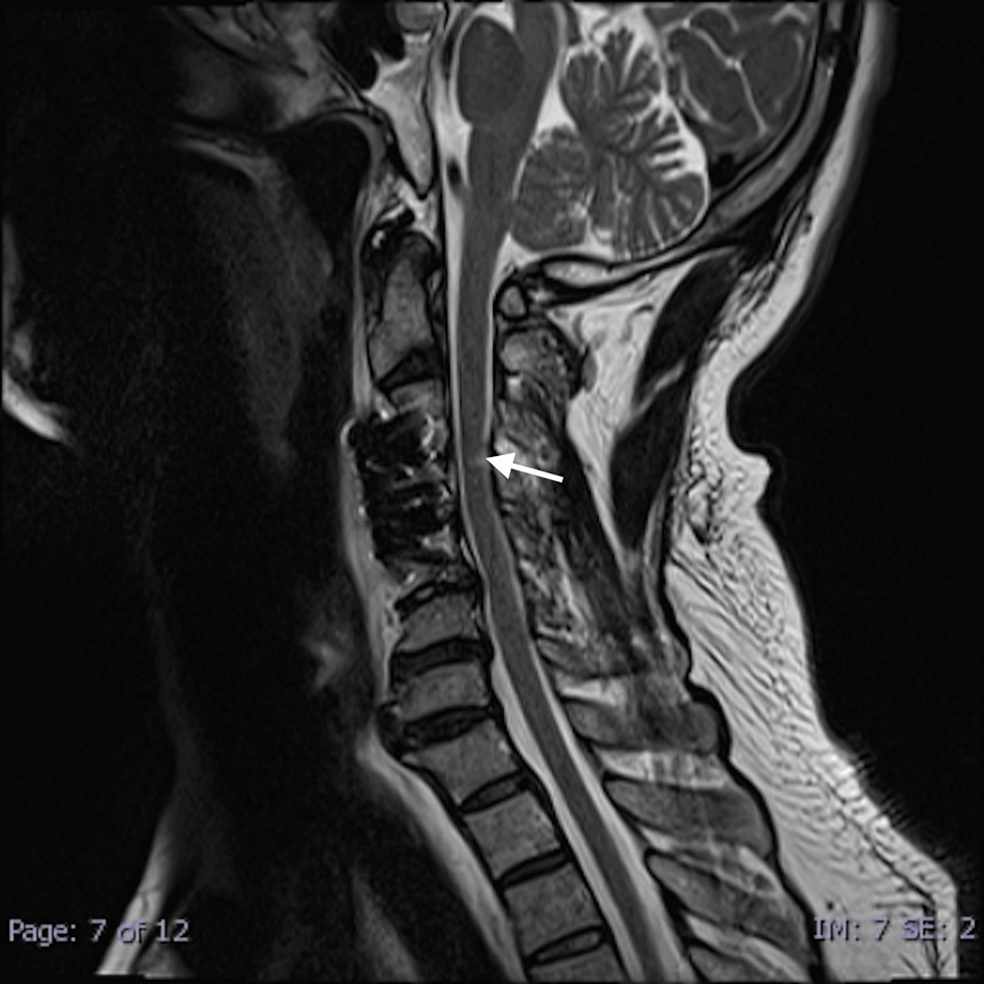
Sagittal T2-Weighted Image Sagittal T2-Weighted MR image of the spine shows a hyperintense signal change on the spinal cord, indicative of white cord syndrome.

By the following morning, the patient developed a weakness, which he described as heaviness, in his lower left extremity. The examination showed left leg monoplegia with diffuse numbness. There was no evidence of a cerebral infarction on the MRI brain. A postoperative MRI of his cervical spine ruled out postoperative hematoma or cord compression but revealed an increase in the T2-hyperintense signal change of the spinal cord (Figures 1, 2). This finding was consistent with an ischemic reperfusion injury of the spinal cord post-decompression, as seen in white cord syndrome.

Treatment included steroids for 72 hours, the avoidance of hypotension, and acute rehab and postoperative physical therapy. One-year follow-up indicated persistent left leg monoplegia and numbness with tibialis anterior strength 2/5. A 15-month follow-up demonstrated tibialis anterior strength of 3/5 in the left leg, indicating improvement.

## Discussion

Degenerative cervical myelopathy (DCM) is the most common progressive, nontraumatic spinal cord injury [6]. The number of patients suffering from DCM is anticipated to rise with the rapidly aging population [7]. The most commonly recommended treatment for DCM is cervical spinal decompression surgery, which is increasingly being performed at ambulatory surgery centers [8].

CSIRI is an extremely rare complication that arises after decompression surgery, typically ACDF [9]. The pathophysiology of CSIRI is unclear, but it is thought to arise from the disruption of the blood-spinal cord barrier, increasing permeability to inflammatory mediators [10]. Additionally, the development of microthrombi occludes the vascular supply to watershed regions of the spinal cord. Altered perfusion occurs due to the internal recoil of the spinal cord after decompression [5].

Chronic compression of the spinal cord causes severe ischemia. After decompression, free oxygen radicals are hypothesized to arrive with reperfusion, potentially driving oxidative stress and injury to the spinal cord [3]. Reactive oxygen radicals also cause lipid peroxidation of the neural membranes [4]. Ischemic-reperfusion injury is thought to arise from this direct trauma to the spinal cord due to the immediate rush of blood after acute decompression [4].

The timing of surgery provides a valuable indication of the severity of the reperfusion injury. A rat model of DCM was utilized to study the effects of early (six weeks) versus delayed (12 weeks) decompression surgery [11]. The study found that delayed surgical decompression used to treat DCM exacerbates reperfusion injury, is associated with ongoing enhanced levels of cytokine expression, microglia activation, and astrogliosis, and results in poorer neurological recovery. Patients with a shorter duration of symptoms (<6 months) exhibited a greater improvement in the mJOA score than those with a longer duration of symptoms (≥6 months) [11].

Since 2013, 19 patients, including ours, have been reported to suffer from CSIRI following cervical decompression for DCM, excluding ossification of the posterior longitudinal ligament, tumors, and trauma. In 73.7% of these patients, the Nurick grade worsened, and 62.3% had poor outcomes. Neurological deficits included tetraplegia (58.3%), paraplegia (16.7%), hemiparesis (16.7%), and monoplegia (8.3%). In 73.7% of patients, the onset of neurological deterioration was immediate (less than one hour postoperatively). To our knowledge, our case represents the first reported instance of lower extremity monoplegia as a manifestation of CSIRI after an ACDF procedure.

A literature search was performed using PubMed, Google Scholar, ScienceDirect, and Cochrane databases. Search terms included (“white cord syndrome” OR “ischemic injury” OR “reperfusion injury”) AND (“anterior cervical decompression” OR “posterior cervical decompression” OR “cervical discectomy”). Any articles in non-English, as well as those including pediatric patients, trauma, tumors, revision surgeries, and non-cervical topics, were excluded. Information regarding age, sex, preoperative myelomalacia, past medical history, surgery performed, deficits, onset, treatment, change in Nurick grade, and outcome.

The literature search yielded 25 articles. After inclusion/exclusion criteria screening, there were 12 articles and 19 total cases reported, including our case.

There were 19 patients (16 males and three females) with a mean age of 61 ± 11.8 years [2,4,5,12-20]. The preoperative Nurick grade averaged 2.53. Specifically, in males, it stood at 2.69, while among females, it was 1.67. Following the operation, the average Nurick grade was 3.33. Males showed an average of 3.47, whereas females averaged 2.67 postoperatively.

Preoperative findings included 14 patients with preoperative myelomalacia, including our patient [4,12-15,17,18,20], 12 patients with hypertension [2,4,5,18,19], four patients with diabetes mellitus [4], one patient with scoliosis/achondroplasia [16], one patient with atrial fibrillation [19], and one patient with heart disease [5].

The surgical approach varied, with 13 patients treated using a posterior approach [4,12,16-20] and six treated with an anterior approach [2,5,13-15]. This distribution resulted in 68.4% of patients developing white cord syndrome having undergone a posterior approach. Seven patients underwent posterior discectomy and fusion surgery [4,12,18,19], while six underwent ACDF [2,5,13-15]. The remaining six patients underwent posterior discectomy [4,16,17,20].

The onset of CSIRI was immediate in 14 patients [2,4,12-14,16,18,20] and delayed in five patients, including ours [5,15,17,19]. Four cases reported a specific time frame for the delay, ranging from one hour to four days postoperation. Postoperative neurological deficits included tetraplegia, identified in seven patients [5,13-17,20], paraplegia, identified in two patients [2,20], hemiparesis, identified in two patients [12,18], and monoplegia, identified only in our case.

Interventions used included steroids in 19 patients [2,4,5,12-20], induced hypertension in five patients [5,12,18,20], and surgery in three patients [2,13,19]. Post-intervention, Nurick’s score worsened in 14 patients [4,12-14,16,17,19], improved in three patients [5,15,18], and remained stable in two patients [2,20]. The long-term outcome based on Nurick grade was poor in 12 patients [4,12-16,19], good in six patients [2,4,5,18,20], and death in one patient [17]. Table 1 summarizes the literature review findings.

**Table 1:** Literature review ACDF: anterior cervical discectomy and fusion; Ach: achondroplasia; AF: atrial fibrillation; DM: diabetes mellitus; HD: heart disease; HTN: hypertension; LE: lower extremity; Lt: left; MAP: mean arterial pressure; ND: no data; PD: posterior decompression; PDF: posterior decompression and fusion; PMHx: past medical history; Pre myel: pre-existing myelopathy; Rt: right; Scol: scoliosis

Article	Age and gender	Pre myel	PMHx	Surgery	Deficits	Onset	Treatment	Δ Nur	Outcome
Jun et al. (2020) [2]	49, Female	-	HTN	ACDF C6-7	Paraplegia	Immediate	Steroids + OR	1 -> 1	Good
Fathalla et al. (2020) [4]	62, Male	+	HTN/DM	PDF C3-7	ND	Immediate	Steroids	2 -> 4	Poor
	65, Male	+	HTN	PDF C3-6	ND	Immediate	Steroids	3 -> 4	Poor
	70, Male	+	HTN/DM	PD C3-7	ND	Immediate	Steroids	3 -> 4	Poor
	61, Male	-	HTN/DM	PDF C3-5	ND	Immediate	Steroids	1 -> 3	Good
	63, Male	+	HTN	PDF C3-6	ND	Immediate	Steroids	3 -> 4	Poor
	69, Female	+	HTN/DM	PD C3-6	ND	Immediate	Steroids	2 -> 4	Poor
	65, Female	+	HTN	PD C3-7	ND	Immediate	Steroids	2 -> 3	Good
Algahtani et al. (2022) [5]	66, Male	-	HTN/HD	ACDF 5-6	Tetraplegia	Delayed	Steroids + MAP	2 -> 1	Good
Antwi et al. (2018) [12]	68, Male	+	ND	PDF C4-7 (3-7)	Lt hemiparesis	Immediate	Steroids + MAP	1 -> 4	Poor
Chin et al. (2013) [13]	59, Male	+	ND	ACDF C4-6	Tetraplegia	Immediate	Steroid + OR	3 -> 4	Poor
Giammalva et al. (2017) [14]	64, Male	+	ND	ACDF C3-4, C5-6	Tetraplegia	Immediate	Steroids	3 -> 4	Poor
Khan et al. (2017) [15]	36, Male	+	None	ACDF C5-6	Tetraplegia	Delayed	Steroids	5 -> 4	Poor
Malinovic et al. (2021) [16]	46, Male	-	Scol/Ach	PD C2-T2	Tetraplegia	Immediate	Steroids	3 -> 5	Poor
Mayoyo and Ouma (2021) [17]	67, Male	+	ND	PD C2-7	Tetraplegia	Delayed	Steroids	3 -> D	Death
Mathkour et al. (2020) [18]	79, Male	+	HTN	PDF C3-5 (2-6)	Rt hemiparesis	Immediate	Steroids + MAP	4 -> 1	Good
Papaioannou et al. (2018) [19]	79, Male	-	HTN/AF	PDF C3-6 (2-7)	Paraplegia	Delayed	Steroids + OR	3 -> 4	Poor
Wiginton et al. (2019) [20]	41, Male	+	ND	PD C1	Tetraplegia	Immediate	Steroids + MAP	1 -> 1	Good
Our patient (2023)	49, Male	+	HTN	ACDF C3-5	Lt LE monopleg	Delayed	Steroids + MAP	3 -> 5	Poor

While the risk factors of CSIRI are not well identified, the following observations can be made from the reviewed cases: 84.2% of patients were male, 68.4% of surgeries used posterior approaches, 73.7% of patients had preoperative myelomalacia, and 85.7% of patients had preoperative hypertension.

## Conclusions

CSIRI, also known as white cord syndrome, is a rare yet devastating complication encountered after cervical spinal decompression surgery. Neurological decline following decompression surgery is typically attributed to direct spinal cord injury, hematoma, or hardware failure; in the absence of these conditions, clinicians must consider the possibility of white cord syndrome. It is believed that this condition arises due to a reperfusion injury affecting the spinal cord, although the precise mechanism is not fully understood. It is postulated that oxidative damage by free radicals may contribute to spinal cord injury. White cord syndrome appears on MRI as a hyperintense signal on T2-weighted images, indicating edema or ischemia in a localized region of the spinal cord. The most common neurological deficits observed in white cord syndrome are paraplegia or paresis. This review provides an overview of CSIRI, including the first reported episode of white cord syndrome characterized by monoplegia. Given the widespread use and benefit of cervical spinal decompression surgery for the treatment of DCM, clinicians should be aware of this potential complication.

## Appendices

Appendix A: Visual Analogue Scale

No pain                                     Moderate pain                                      Worst pain 

    0      1          2          3          4          5          6          7          8          9          10

Appendix B: Neck Disability Index (NDI)

Section 1: Pain Intensity

I have no pain at the moment.

The pain is very mild at the moment.

The pain is moderate at the moment.

The pain is fairly severe at the moment.

The pain is very severe at the moment.

The pain is the worst imaginable at the moment.

Section 2: Personal Care (Washing, Dressing, etc.)

I can look after myself normally without causing extra pain.

I can look after myself normally but it causes extra pain.

It is painful to look after myself and I am slow and careful.

I need some help but I can manage most of my personal care.

I need help every day in most aspects of self-care.

I do not get dressed; I wash with difficulty and stay in bed.

Section 3: Lifting

I can lift heavy weights without extra pain.

I can lift heavy weights but it gives extra pain.

Pain prevents me from lifting heavy weights off the floor, but I can manage if they are conveniently placed, for example on a table.

Pain prevents me from lifting heavy weights but I can manage light to medium weights if they are conveniently positioned.

I can only lift very light weights.

I cannot lift or carry anything.

Section 4: Reading

I can read as much as I want to with no pain in my neck.

I can read as much as I want to with slight pain in my neck.

I can read as much as I want with moderate pain in my neck.

I can’t read as much as I want because of moderate pain in my neck.

I can hardly read at all because of severe pain in my neck.

I cannot read at all

Section 5: Headaches

I have no headaches at all.

I have slight headaches, which come infrequently.

I have moderate headaches, which come infrequently.

I have moderate headaches, which come frequently.

I have severe headaches, which come frequently.

I have headaches almost all the time.

Section 6: Concentration

I can concentrate fully when I want to with no difficulty.

I can concentrate fully when I want to with slight difficulty.

I have a fair degree of difficulty concentrating when I want to.

I have a lot of difficulty in concentrating when I want to.

I have a great deal of difficulty in concentrating when I want to.

I cannot concentrate at all.

Section 7: Work

I can do as much work as I want to.

I can only do my usual work, but no more.

I can do most of my usual work, but no more.

I cannot do my usual work.

I can hardly do any work at all.

I can’t do any work at all.

Section 8: Driving

I can drive my car without any neck pain.

I can drive my car as long as I want with slight pain in my neck.

I can drive my car as long as I want with moderate pain in my neck.

I can’t drive my car as long as I want because of moderate pain in my neck.

I can hardly drive at all because of severe pain in my neck.

I can’t drive my car at all.

Section 9: Sleeping

I have no trouble sleeping.

My sleep is slightly disturbed (less than one hour sleepless).

My sleep is mildly disturbed (one to two hours sleepless).

My sleep is moderately disturbed (two to three hours sleepless).

My sleep is greatly disturbed (three to five hours sleepless).

My sleep is completely disturbed (five to seven hours sleepless).

Section 10: Recreation

I am able to engage in all my recreation activities with no neck pain at all.

I am able to engage in all my recreation activities, with some pain in my neck.

I am able to engage in most, but not all of my usual recreational activities because of pain in my neck.

I am able to engage in a few of my usual recreation activities because of the pain in my neck.

I can hardly do any recreational activities because of the pain in my neck.

I can’t do any recreational activities at all.

Score:    /50 

Transform to percentage score x 100 = % points

Scoring: For each section, the total possible score is 5: if the first statement is marked the section score = 0, if the last statement is marked it = 5. If all ten sections are completed the score is calculated

Minimum Detectable Change (90% confidence): 5 points or 10% points

Appendix C: Modified Japanese Orthopaedic Association Scale

Upper Extremity Motor Dysfunction

0 = Unable to move

1 = Unable to feed oneself

2 = Able to feed with great difficulty

3 = Able to feed with slight difficulty

4 = Normal with slight slowing

5 = Normal

Lower Extremity Motor Dysfunction

0 = Complete loss of motor and sensory function

1 = Sensation preserved, no movement

2 = Able to move, but unable to walk

3 = Able to walk on flat ground with a cane or aid

4 = Able to walk up and/or down stairs with aid of a handrail

5 = Moderate to severe lack of stability but able to walk up and/or down stairs without aid of a handrail

6 = Mild lack of stability, no difficulty in walking up and/or down stairs

7 = Normal

Sensory Dysfunction

0 = Complete loss of hand sensation

1 = Severe sensory loss or pain

2 = Mild sensory loss

3 = Normal

Bladder Dysfunction

0 = Unable to void

1 = Marked difficulty in micturition

2 = Mild to moderate difficulty in micturition

3 = Normal
